# The Use of Purgatives in Appendicitis

**Published:** 1907-06

**Authors:** 


### THE USE OF PURGATIVES IN APPENDICITIS.

While the operative technic in appendicitis may be considered as well-nigh perfect, there is still much left to be learned in regard to its diagnosis. Many a diagnosis error still discredits surgery, and too frequent resort to exploratory operations must be considered a confession of weakness. It will be remembered that but recently Dieulafoy showed that certain forms of inflammation of the cecum and colon, especially membranous typhlocolitis, may give rise to symptoms closely resembling appendicitis, and that failure to recognize these cases may lead to useless operation—useless, at least, in the sense that the patient is not benefited. There is abundant reason to believe that in many cases of colitis the appendix is more or less affected, and the question here is to determine whether this involvement is sufficient to make the case a surgical one.

It is therefore of interest to note that Professor E. Sonnenburg, the eminent Berlin surgeon, confesses the difficulty sometimes experienced even by a skilled diagnostician in differentiating between appendicitis and gastro-enteritis, especially if the latter occurs in the course of some general infection, as an influenza. In an article in the Deutsche Medicinische Wochenschrift, April 4, he points out that an increased tenderness in the ileocecal region, together with severe general symptoms, is commonly present in such cases, though operation may show no inflammation of the appendix or peritoneum. It is, of course, possible for a true appendicitis to complicate an enterocolitis, especially if the appendix is the seat of previous inflammatory changes, but these cases present a very characteristic symptomatology and are quite rare.

In many a case of enterocolitis resembling appendicitis the very important question will arise as to the advisability of purgation to free the bowel of irritating material, which would be the logical thing to do whenever putrefactive processes are suspected. Owning to the general teaching that it is unwise, or even reprehensible, to administer purgatives in any condition resembling appendicitis, many physicians are deterred from adopting such treatment. Professors Sonnenburg, however, does not share this fear, and tells us that in doubtful cases in which it is difficult to differentiate between enterocolitis and appendicitis it is often proper to unload the bowel. He sees no objection to giving a dose of castor oil to such patients, provided no gastric disturbances exist, and believes that if the surgeon carefully watches the case, prepared to operate at once if the ptient becomes worse, the risk of perforation or gangrene from the use of purgatives may be practically disregarded; in fact, he thinks that, should surgical intervention be contemplated, an empty intestine would be desirabe in the post-operative treatment. Recently in cases of dificut diagnosis as well as in the early stage of appendicitis he has given castor oil, making at the same time all preparations for an immediate operation if deemed necessary. By pursuing such a policy it has been often possible to avoid abdominal section.

Of course, this does not mean the indiscriminate administration of purgatives in appendicitis, for in severe cases there is only one remedy—the knife as early as possible; but where the diagnosis is doubtful and the indications call for tmptying the bowel, the physicians should not be deterred by the unwarranted fear of purgation. From this point of view Professos Sonnenburg's contribution, based as it is upon an immense experience in the treatment of appendicitis, is deserving of careful consideration—Ed. Internat. Jour. Surg., May, 1907.

